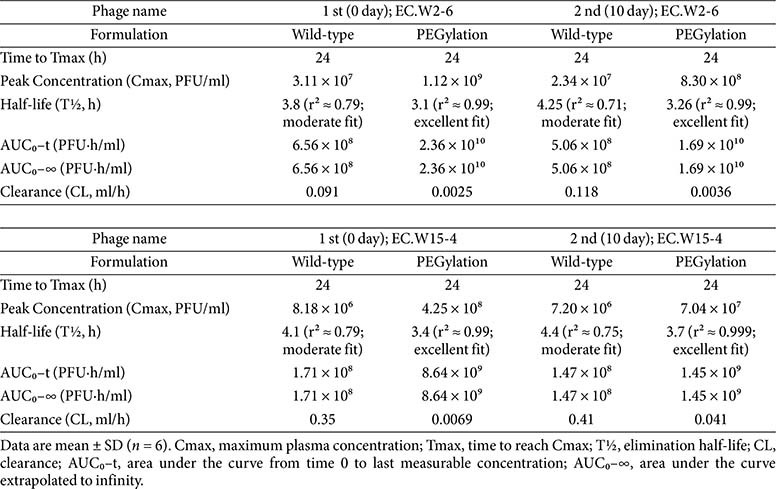# PEGylation Overcomes Pharmacological Barriers to Improve Systemic Pharmacokinetics and Therapeutic Efficacy of Phages against MDR *Escherichia coli*

**DOI:** 10.4014/jmb.2509.09050

**Published:** 2025-11-26

**Authors:** Md Shamsuzzaman, Yoon-Jung Choi, Shukho Kim, Ji Yun Jeong, Cheol Am Hong, Jungmin Kim

**Affiliations:** 1 Department of Microbiology, School of Medicine, Kyungpook National University, Daegu 41944, Republic of Korea; 2 Untreatable Infectious Disease Institute, Kyungpook National University, Daegu 41944, Republic of Korea; 3 Department of Chemistry, College of Natural Sciences, Yeungnam University, Gyeongsan-si 38541, Republic of Korea

**Keywords:** Phage therapy, PEGylation, pharmacokinetics, *Escherichia coli*, multidrug resistance, *In vivo* infection model

## Abstract

Systemic bacteriophage therapy against multidrug-resistant (MDR) *Escherichia coli* is fundamentally limited by rapid immune-mediated clearance, complement activation, and phagocytic sequestration, collectively constituting pharmacological barriers that restrict systemic bioavailability, shorten circulation half-life, and attenuate therapeutic efficacy. We hypothesized that PEGylation, by sterically shielding phage capsids from host immune clearance mechanisms, would enhance systemic stability, improve pharmacokinetic (PK) behavior, and augment therapeutic efficacy *in vivo*. Four lytic *E. coli* phages were covalently conjugated with 5-kDa mPEG-S-NHS, achieving >60% surface amine modification as confirmed by fluorescamine assay. PEGylation resulted in a ~1.5–5 log_10_ reduction in infectious titer and modestly slowed adsorption kinetics but preserved latent period and burst size, confirming intact replication competence. In serum, wild-type phages were undetectable within 24–48 h, whereas PEGylated phages retained ~2–3 log_10_ PFU ml^−1^ at 24 h and persisted longer within RAW264.7 macrophages and HT-29 epithelial cells. In mice, PEGylation markedly increased systemic exposure (AUC_0_–∞ up to 50-fold), prolonged circulation, and reduced clearance >15-fold. In infected hosts, PEG-EC.W2-6 and PEG-EC. W15-4 achieved plasma titers up to 100-fold higher with >30-fold lower clearance, accelerating bacterial elimination (72 h vs 96 h). Despite partial IgG induction upon repeated dosing, PEGylated phages maintained superior PK and significantly suppressed infection-driven IL-6, IFN-γ, TNF-α, and IL-1β, normalizing cytokine profiles toward baseline. Overall, PEGylation markedly improves systemic persistence, intracellular stability, and immunomodulatory efficacy, representing a robust strategy to overcome PK barriers and optimize systemic phage therapy against MDR *E. coli*.

## Introduction

The global rise of MDR bacterial infections poses an urgent and escalating threat to human health. *Escherichia coli* is ranked among the World Health Organization’s critical-priority Gram-negative pathogens due to its increasing resistance to carbapenems and other last-resort antibiotics [1]. The limited pipeline of novel antimicrobials has reinvigorated interest in phage therapy, which offers precision targeting of bacterial pathogens while sparing the commensal microbiota [2, 3]. Despite promising case reports and small clinical studies, translation of phage therapy to systemic applications has been hampered by a fundamental pharmacological challenge the rapid clearance of exogenously administered phages from circulation [4-6].

Following intraperitoneal delivery, phages are rapidly removed by innate and adaptive immune mechanisms. Complement-mediated inactivation, opsonization, and phagocytosis by the reticuloendothelial system can eliminate circulating phages within minutes [7, 8]. In this context, pharmacological barriers refer to the host physiological and immunological defense mechanisms, such as complement inactivation, opsonization, and phagocytic sequestration that limit systemic bioavailability, reduce circulation half-life, and constrain the therapeutic performance of phages [9]. Long-circulating phage variants have been reported, while repeated administration accelerates neutralization through the induction of anti-phage antibodies [10, 11]. These barriers limit systemic bioavailability, reduce therapeutic exposure at infection sites, and undermine efficacy in disseminated infections and sepsis models [12]. Indeed, several recent clinical trials have highlighted poor PK as a major reason for limited success in systemic phage therapy [13, 14].

To overcome these barriers, multiple strategies have been explored. Genetic engineering of phage capsids can reduce recognition by immune receptors [15], and adaptive evolution *in vivo* may select for variants with prolonged circulation [9]. However, these approaches are technically demanding, often phage-specific, and may compromise host range or stability. By contrast, biochemical surface modification offers a broadly applicable and scalable method to improve systemic persistence without altering phage genomes [16].

PEGylation is a clinically validated strategy to improve the PK of protein and nanoparticle therapeutics [17]. PEG provides a hydrophilic steric barrier that reduces recognition by complement proteins, antibodies, and phagocytic receptors, thereby extending circulation half-life as demonstrated by PEGylated therapeutics already used in the clinic [18]. PEGylation has been adapted to viral vectors and more recently to phages, where it has shown potential to reduce immune clearance and prolong systemic exposure [19]. However, clinical translation remains limited, in part because optimal PEGylation parameters—balancing immune shielding with preservation of adsorption efficiency and lytic activity—are not well defined. Previous studies reported that excessive PEGylation can impair infectivity [9], underscoring the need for systematic evaluation across diverse phages.

In this study, we systematically evaluated PEGylation in four lytic *E. coli* phages with diverse structural and functional properties. Using fluorescamine-based quantification, we achieved >60% surface amine conjugation and assessed effects on adsorption kinetics, burst size, and replication competence. This study further compared serum stability, intracellular persistence, and PK in both infected and non-infected murine models, alongside profiling immune responses. Our findings demonstrate that optimized PEGylation markedly improves systemic persistence, enhances intracellular stability, accelerates bacterial clearance, and reduces inflammatory cytokines, while inducing weaker anti-phage antibody responses compared with wild type. This work highlights PEGylation as a practical, reproducible, and broadly applicable strategy to overcome the PK barriers of systemic phage therapy and advance therapeutic development against MDR *E. coli* infections.

## Methods

### Bacterial Strains and Phage Preparation

Two *E. coli* strains were selected for this study: ATCC 25922 (reference strain) and KBN10P07288, a clinically isolated carbapenem-resistant strain obtained from Kyungpook National University Hospital Korea. Both strains were routinely cultured in brain heart infusion (BHI) broth and agar media at 37°C and preserved at –70°C in 20%glycerol stocks for long-term use.

Four lytic *E. coli* phages (EC.W2-6, EC.W13-3, EC.W15-3, EC.W15-4) were propagated on ATCC 25922 [20]. Mid-log cultures (OD_600_ ≈ 0.5; ~1 × 10^8^ CFU/ml) were infected at a multiplicity of infection (MOI) of 0.1 and incubated overnight at 30°C. Lysates were clarified by centrifugation (12,000 ×*g*, 10 min, 4°C), filtered through 0.22 μm membranes, and concentrated to ~10^12^–10^13^ PFU/ml using 100 kDa centrifugal filters (Millipore, Germany). Phages were suspended in 10 mM sodium phosphate buffer (pH 7.5) and stored at 4°C.

### PEGylation and *In Vitro* Characterization

Purified phages (≥ 1 × 10^13^ PFU/ml) were reacted with 5 kDa methoxy polyethylene glycol succinimidyl ester (mPEG-S-NHS; Sigma-Aldrich, USA) at 25°C for 12 h under gentle agitation at an approximate phage-to-PEG molar ratio of 1:5,000. The NHS ester reacts with surface-exposed lysine residues and terminal amines on the capsid to form stable amide linkages. Unreacted PEG was removed by ultrafiltration (10 kDa cut-off; Sartorius) and samples were washed three times with nuclease-free water. PEGylation efficiency was determined by fluorescamine assay, which detects residual free amine groups on phage capsids. PEGylation was considered saturated when additional PEG did not further reduce fluorescence (Figs. S1–S3) [17]. Independent conjugations performed in three batches yielded comparable amine reductions (~60 ± 5%) and similar PFU decreases (2–4 log_10_), confirming reproducibility. Although this approach does not quantify absolute grafting density or PEG distribution, it effectively compares modification levels across samples.

Phage activity was evaluated using plaque assays, adsorption kinetics, and one-step growth curves. For adsorption assays, exponentially growing *E. coli* ATCC 25922 cultures were infected at MOI of 0.0001, and unabsorbed phages were quantified at defined time intervals [20]. One-step growth experiments were performed by allowing phages to adsorb at 4°C for 30 min to synchronize infection, followed by incubation at 37°C. Samples were collected every 5 min for 60 min to determine latent period and burst size [20]. All experiments were performed in triplicate.

### Serum and Intracellular Stability

Serum stability was measured by mixing phages (1 × 10^8^ PFU/ml) with heat-inactivated mouse and human serum (1:1) and incubating at 37°C. Infectious titers were quantified by plaque assay at 0, 1, 2, 4, 8, 24, and 48 h [19]. Intracellular stability was assessed in RAW 264.7 macrophages (ATCC TIB-71) and HT-29 epithelial cells (KCLB Pass-K33). Cells (1 × 10^6^/well, 24-well plates) were infected with phages at 1 × 10^7^ PFU/well (MOI ≈ 10) and incubated for 24 h at 37°C. Before cell lysis, monolayers were washed three times with sterile PBS to remove unbound or surface-adherent phages. Cells were then lysed with 0.1% Triton X-100, centrifuged (≈7,000 ×*g*, 20 min), filtered (0.2 μm), and treated with 10 % chloroform. Surviving intracellular phages were quantified by plaque assay. Although PBS washing minimizes extracellular contamination, we acknowledge that Triton X-100 lysis may not completely exclude surface-bound phages.

### *In Vivo* Efficacy and PK Studies

Among the four isolated phages (three *Straboviridae* and one *Gordonclarkvirinae*), EC.W2-6 (*Gordonclarkvirinae*, genus *Kuravirus*) and EC.W15-4 (*Straboviridae*, genus *Tequatrovirus*) were selected for *in vivo* evaluation based on their distinct structural lineages, broad host range, and superior stability under physiological conditions. All animal experiments were approved by the Institutional Animal Care and Use Committee of Kyungpook National University (Approval No. KNU-2023-0478). Female BALB/c mice (6–8 weeks old, n =6 per group) received intraperitoneal injections of wild-type or PEGylated phages EC.W2-6 and EC.W15-4 (2.24 × 10^8^ PFU in 200 μl) as repeated doses on Day 0 and Day 10. For infection experiments, mice were challenged intraperitoneally with *E. coli* KBN10P07288 (1 × 10^8^ CFU in 200 μl) and treated with the same repeated phage dosing regimen (Day 0 and Day 10), with the first dose administered 30 min after bacterial challenge. Blood samples were collected at 1, 2, 4, 8, 24, 48, 72, and 120 h post-injection. Plasma phage titers were quantified by plaque assay, and bacterial counts determined by colony enumeration [21, 22].

PK parameters—including maximum plasma concentration (Cmax), time to maximum concentration (Tmax), area under the plasma concentration–time curve (AUC), half-life (T½), and systemic clearance (CL)—were calculated by non-compartmental analysis using Phoenix WinNonlin (Certara). AUC was estimated by the linear trapezoidal method, and T½ derived from the terminal elimination constant (Kel).

### Immune Response Assays

Anti-phage IgG responses were measured by ELISA. Briefly, purified phages (0.6 μg/well) were immobilized on 96-well plates, blocked with 3% bovine serum albumin (BSA), and incubated with serum samples (1:400 dilution). Bound IgG was detected using alkaline phosphatase–conjugated anti-mouse IgG (1:5,000), and absorbance was recorded at 405 nm after 20 min using p-nitrophenyl phosphate (pNPP) substrate.

### Serum Cytokine Profiling

Serum IL-6, IFN-γ, TNF-α, and IL-1β were quantified using a mouse cytokine multiplex ELISA kit (Manufacturer, Cat: ARG82842) according to the manufacturer’s protocol. Blood was collected 24 h post-treatment, clotted at room temperature, and centrifuged at 3,000 ×*g* for 10 min to isolate serum. Samples (50–100 μl) were added to pre-coated plates, incubated with biotinylated detection antibodies and streptavidin–HRP, and developed with TMB substrate. Absorbance was measured at 450 nm using a VersaMax PLUS microplate reader. Cytokine concentrations were calculated from standard curves and expressed as pg/ml. Each group consisted of *n* = 3 mice, and all data represent mean ± SD of technical triplicates.

### Statistical Analysis

All experiments were performed with at least three independent biological replicates. For serum cytokine profiling, *n* = 3 mice per group were used. Data are reported as mean ± standard deviation (SD). Statistical significance was determined using unpaired two-tailed Student’s *t*-tests (for two-group comparisons) or one-way ANOVA with Tukey’s post hoc test (for multiple-group comparisons) in GraphPad Prism (version 10.2; GraphPad Software, USA). A *p*-value < 0.05 was considered statistically significant.

## Results

### PEGylation Efficiency and Phage Viability

**Fig. 1:**
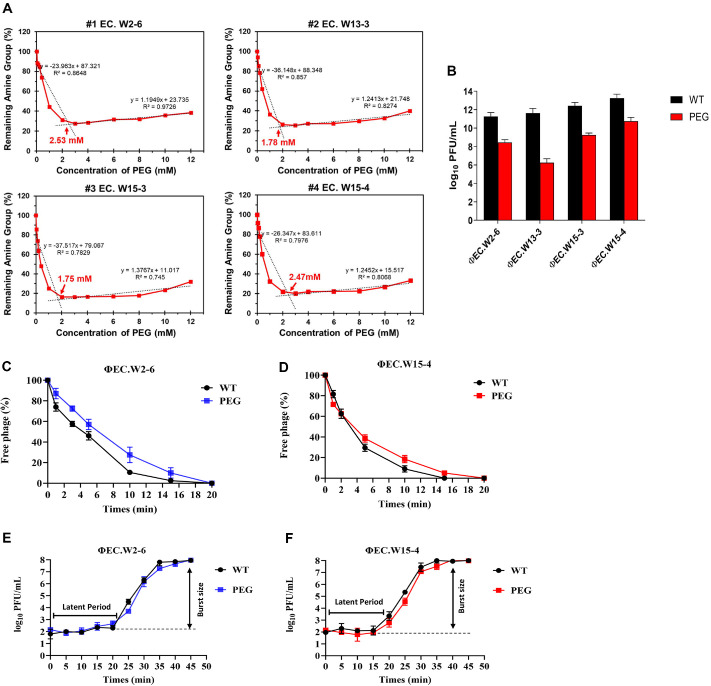
PEGylation efficiently modified the surface of *E. coli* phages, as confirmed by the fluorescamine assay (A) showing a concentrationdependent decrease in fluorescence and saturation at 1.75–2.53 mM, indicating >60% reduction in surface-exposed amines. Infectious titers decreased by ~1.5–5 log_10_ following PEGylation (**B**) likely due to partial steric interference with receptor-binding proteins. Adsorption kinetics were modestly delayed, with >80% of PEGylated phages attaching within 10 minutes compared to ~90% for wild type (**C–D**). Despite slower adsorption, one-step growth curves revealed no significant differences in latent period (~20 min) or burst size (**E–F**), indicating that PEGylation preserves phage replication and lytic capacity.

Fluorescamine assays confirmed efficient PEGylation of surface-exposed amines in all four *E. coli* phages, showing a concentration-dependent fluorescence decline. Saturation occurred at 2.53 mM for PEG-EC.W2-6, 1.78 mM for PEG-EC.W13-3, 1.75 mM for PEG-EC.W15-3, and 2.47 mM for PEG-EC.W15-4, corresponding to > 60% reduction in accessible amines relative to unmodified controls (Fig. 1A).

PEGylation led to a ~1.5–5 log_10_ decrease in infectious titers (Fig. 1B), attributed to partial steric masking of receptor-binding proteins (RBPs) rather than structural damage, since latent period and burst size were unchanged. Adsorption kinetics were modestly delayed, with > 80 % of PEGylated phages adsorbing within 10 min versus ~90% for wild type (Fig. 1C and 1D), while replication competence remained intact (Fig. 1E and 1F). These findings indicate that PEGylation partially shields RBPs yet preserves productive infection, consistent with previous observations

### Serum Stability and Intracellular Persistence of PEGylated Phages

**Fig. 2:**
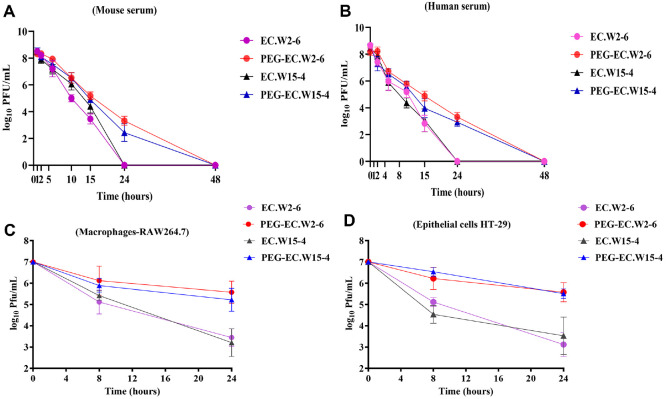
PEGylation enhances phage stability in serum and host cells. (**A–B**) In both mouse (**A**) and human (**B**) serum, PEGylated EC.W2-6 and EC.W15-4 maintained titers of ~2–3 log_10_ PFU/ml at 24 h, whereas wild-type phages were rapidly inactivated and fell below detection limits. (**C–D**) In RAW264.7 macrophages (**C**) and HT-29 epithelial cells (**D**), PEGylated phages exhibited significantly higher intracellular persistence (~5–6 log_10_ PFU/ml at 24 h) compared to wild type (~2–4 log_10_ PFU/ml). Data are presented as mean ± SD from three biological replicates.

Serum stability assays demonstrated that wild-type EC.W2-6 was rapidly inactivated in mouse serum and became undetectable within 24 h, while wild-type EC.W15-4 declined more gradually but was completely cleared by 48 h. In contrast, PEGylation significantly enhanced phage persistence, with PEG-EC.W2-6 and PEG-EC.W15-4 retaining approximately 3 log_10_ PFU/ml after 24 h of incubation (Fig. 2A). Similar trends were observed in human serum, where both wild-type phages dropped below the detection threshold by 24 h, whereas PEGylated phages maintained titers of ~2–3 log_10_ PFU/ml, with PEG-EC.W2-6 exhibiting slightly higher levels than PEG-EC.W15-4 (Fig. 2B).

Intracellular persistence assays corroborated these findings. In RAW264.7 macrophages, wild-type phages declined to ~3–4 log_10_ PFU/ml at 24 h, while PEGylated phages maintained significantly higher titers of ~5–6 log_10_ PFU/ml (Fig. 2C). Similarly, in HT-29 epithelial cells, PEG-EC.W2-6 and PEG-EC.W15-4 sustained intracellular titers of ~5–6 log_10_ PFU/ml, compared to only ~2–3 log_10_ PFU/ml for their wild-type counterparts at the same time point (Fig. 2D).

### PEGylation Enhances PK and Systemic Persistence of *E. coli* Phages in Non-Infected Mice

**Fig. 3:**
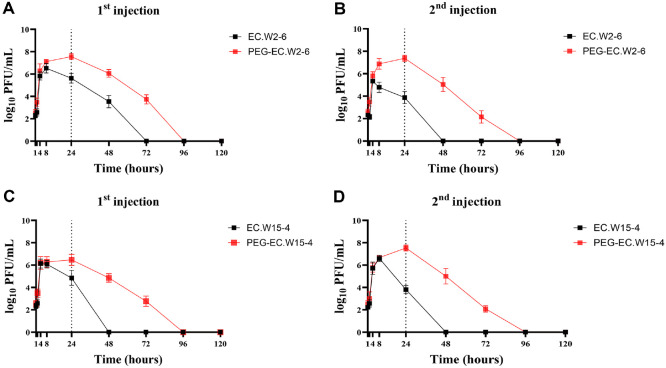
PEGylation improves phage PK in mice. PK profiles of wild-type and PEGylated EC.W2-6 (top) and EC.W15-4 (bottom) after the first (left) and second (right) intraperitoneal injections. PEGylated phages exhibited delayed Tmax, higher Cmax, prolonged circulation, and markedly reduced clearance compared with wild type. Data represent mean ± SD (*n* = 6 per group).

PK analysis demonstrated that PEGylation significantly enhanced the systemic persistence of *E. coli* phages following intraperitoneal administration in non-infected mice (Fig. 3; Table 1). After a single injection, wild-type EC.W2-6 reached a maximum plasma concentration (Cmax) of 3.29 × 10^6^ PFU/ml at 8 h, with a half-life of 4.76 h and an area under the curve (AUC_0_–∞) of 5.82 × 10^7^ PFU·h/ml. In contrast, PEG-EC.W2-6 exhibited a delayed Tmax of 24 h, a significantly higher Cmax of 3.66 × 10^7^ PFU/ml (~11-fold increase), and a ~17-fold increase in AUC_0_–∞ (1.01 × 10^9^ PFU·h/ml). This was accompanied by a >15-fold reduction in systemic clearance (0.059 vs 1.03 ml/h).

A similar PK enhancement was observed for PEG-EC.W15-4. While the wild-type EC.W15-4 peaked at 1.75 × 10^6^ PFU/ml (Tmax 4 h) with an AUC_0_–∞ of 2.21 × 10^7^ PFU·h/ml, PEGylation resulted in a delayed Tmax (24 h), a modestly increased Cmax of 4.04 × 10^6^ PFU/ml, and a dramatically improved AUC_0_–∞ of 1.19 × 10^9^ PFU·h/ml —representing a nearly 54-fold increase. Clearance was also substantially reduced (0.50 vs 2.72 ml/h).

Following repeat administration on Day 10, systemic exposure declined relative to the initial dose, likely reflecting partial induction of anti-phage immunity. Nevertheless, PEG-EC.W2-6 and PEG-EC.W15-4 continued to demonstrate superior PK, maintaining Cmax values of 2.67 × 10^7^ and 3.78 × 10^7^ PFU/ml, respectively. Their AUC_0_–∞ values remained high (6.57 × 10^8^ and 8.10 × 10^8^ PFU·h/ml), while the wild-type phages were rapidly eliminated, showing Cmax ≤ 4.51 × 10^5^ PFU/ml and AUC_0_–∞ ≤ 4.79 × 10^7^ PFU·h/ml.

### PEGylation Enhances PK and Therapeutic Efficacy of *E. coli* Phages in Infected Mice

**Fig. 4:**
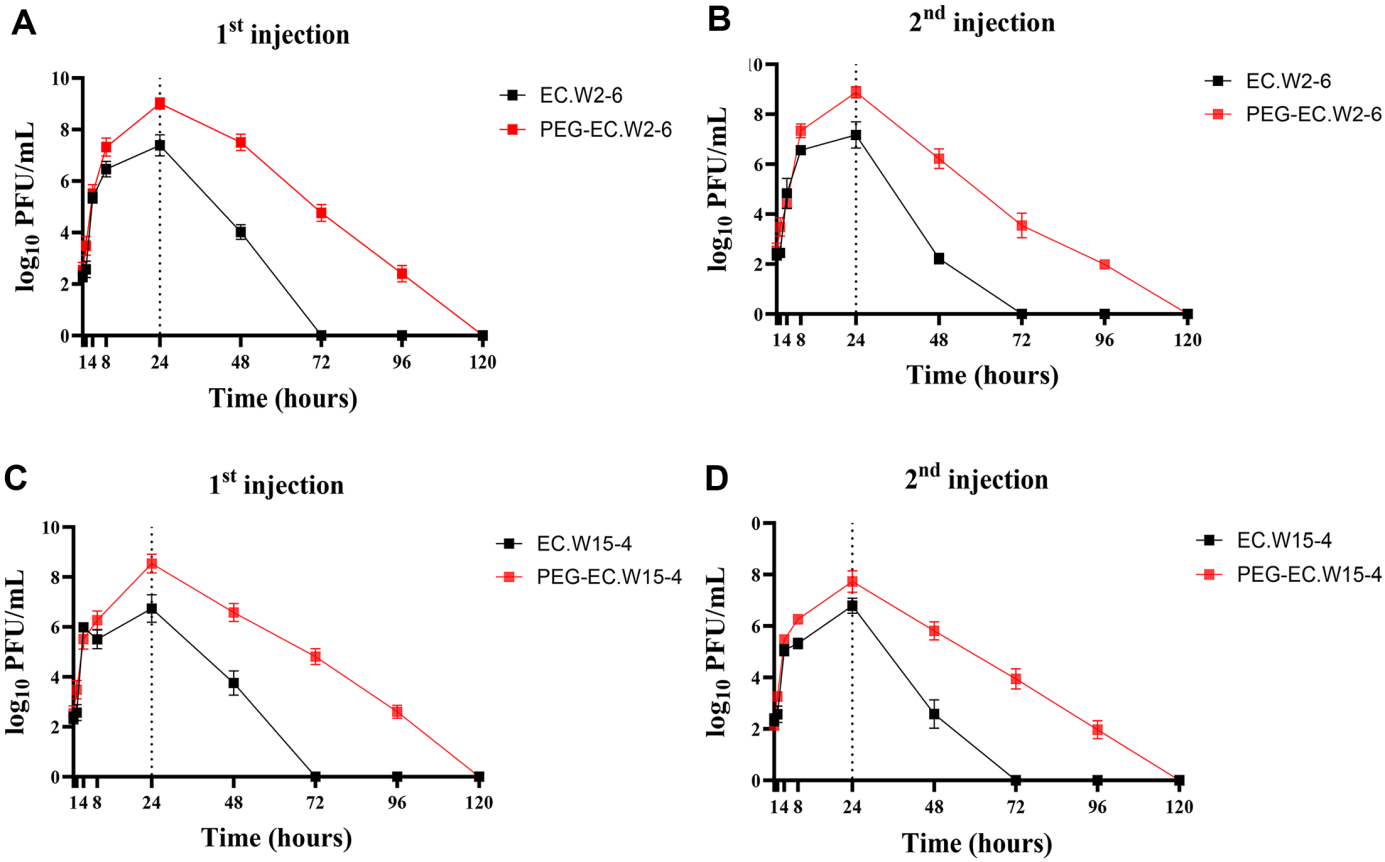
PEGylation enhances phage PK and bacterial clearance in infected mice. (**A–D**) Serum PK of EC.W2-6 (A–B) and EC.W15-4 (**C–D**) after the first (left) and second (right) intraperitoneal injections in infected mice. PEGylated phages exhibited delayed Tmax, higher Cmax, prolonged circulation, and significantly increased AUC compared with wild type. (**E–H**) Blood bacterial burden following treatment. PEGylated phages achieved significantly greater clearance of circulating bacteria, reflected by reduced CFU counts after both injections. Data represent mean ± SD (*n* = 6 per group).

PK profiling in infected mice revealed that PEGylation significantly improved the systemic persistence of both EC.W2-6 and EC.W15-4 in the presence of their bacterial host (*E. coli* KBN10P07288) (Fig. 4A–4H; Table 2). Following a single intraperitoneal injection, wild-type EC.W2-6 achieved a peak plasma concentration (Cmax) of 3.11 × 10^7^ PFU/ml at 24 h, with a half-life of 3.8 h and an area under the curve (AUC_0_–∞) of 6.56 × 10^8^ PFU·h/ml. In comparison, PEG-EC.W2-6 reached a substantially higher Cmax of 1.12 × 10^9^ PFU/ml, while maintaining the same Tmax (24 h), and showed a ~36-fold increase in systemic exposure (AUC_0_–∞ = 2.36 × 10^10^ PFU·h/ml) along with a >30-fold reduction in clearance (0.0025 vs 0.091 ml/h).

PEG-EC.W15-4 exhibited similar enhancements. PEGylation raised the Cmax from 8.18 × 10^6^ to 4.25 × 10^8^ PFU/ml (~52-fold), increased the AUC_0_–∞ ~50-fold (8.64 × 10^9^ vs 1.71 × 10^8^ PFU·h/ml), and decreased systemic clearance nearly 50-fold (0.0069 vs 0.35 ml/h).

After repeat dosing on Day 10, PEG-EC.W2-6 and PEG-EC.W15-4 retained strong PK advantages over their wild-type counterparts despite evidence of partial humoral response. Cmax values remained high (8.30 × 10^8^ and 7.04 × 10^7^ PFU/ml, respectively), and AUC_0_–∞ values remained approximately 10-fold greater than wild-type phages.

Importantly, the improved PK of PEGylated phages translated into superior therapeutic efficacy. Mice treated with PEG-EC.W2-6 or PEG-EC.W15-4 achieved complete bacterial clearance within 72 h, compared to 96 h for wild-type phages. This accelerated clearance was sustained following the second dose, with PEGylated phage-treated groups maintaining significantly lower bacterial loads over the full 120 h observation period. No clinical signs of toxicity, weight loss, or mortality were observed in any treatment group, indicating that both PEGylated and wild-type phages were well tolerated *in vivo*.

### Antibody IgG Response

**Fig. 5:**
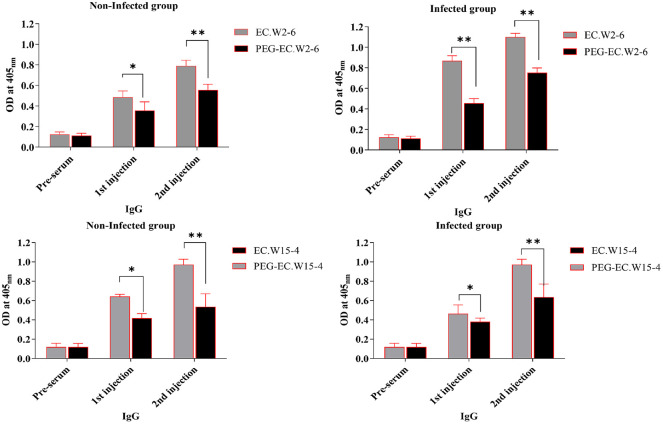
Anti-phage IgG responses in non-infected and infected mice. Mice received two intraperitoneal injections (on Day 0 and Day 10) of wild-type or PEGylated EC.W2-6 and EC.W15-4. Sera were collected 10 days after each injection and analyzed by ELISA. Data are presented as mean ± SD (*n* = 3 per group). Statistical analysis was performed using one-way ANOVA with Tukey’s post hoc test. *p < 0.05, **p < 0.01, ***p < 0.001 vs wild-type.

The anti-phage IgG response was assessed using ELISA in both non-infected and infected mice following two consecutive administrations of either wild-type or PEGylated phages EC.W2-6 and EC.W15-4 (Fig. 5). In non-infected mice, wild-type formulations elicited significantly higher IgG responses than their PEGylated counterparts (*p* < 0.05 or *p* < 0.01). For EC.W2-6, mean OD values increased from a baseline of 0.12 to 0.49 after the first injection and 0.79 following the second. In contrast, PEG-EC.W2-6 induced lower OD values of 0.36 and 0.56, respectively. A comparable trend was observed for EC.W15-4, with wild-type OD values rising to 0.64 and 0.97, whereas PEG-EC.W15-4 induced weaker responses (0.42 and 0.54).

In infected mice, overall IgG responses were higher, consistent with enhanced immune stimulation in the presence of bacterial infection. Wild-type EC.W2-6 triggered OD values of 0.87 and 1.10 after the first and second injections, respectively. However, PEGylated EC.W2-6 induced significantly attenuated responses of 0.46 and 0.75 (*p* < 0.01). Similarly, wild-type phage EC.W15-4 elicited responses of 0.46 and 0.97, compared to 0.38 and 0.64 for PEG-EC.W15-4. These results clearly demonstrate that PEGylation significantly attenuates anti-phage IgG responses, supporting improved PK and systemic persistence in both non-infected and infected hosts.

### Cytokine Analysis

**Fig. 6:**
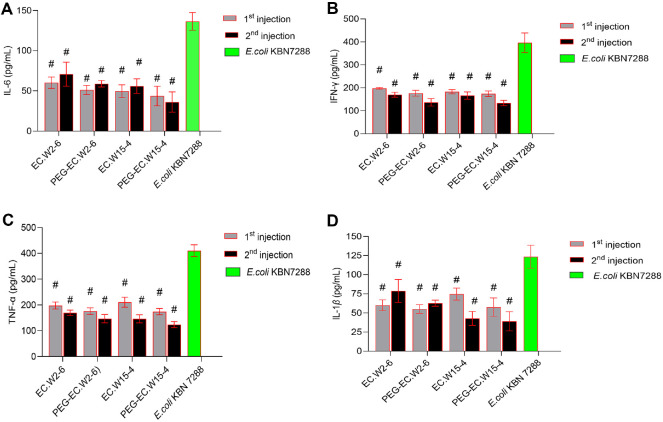
PEGylated phages attenuate infection-induced cytokine responses. BALB/c mice infected with *E. coli* KBN10P07288 were treated with wild-type or PEGylated phages (EC.W2-6 and EC.W15-4). Serum cytokines (IL-6, IFN-γ, TNF-α, and IL-1β) were measured 24 h after the first (Day 0) and second (Day 10) injections using multiplex ELISA. PEGylated phages showed stronger cytokine suppression than wild-type phages. Data are mean ± SD (*n* = 3); significance was analyzed by one-way ANOVA (*p* < 0.05). #: significantly different from the *E. coli* KBN7288 group.

Quantitative cytokine analysis confirmed that systemic infection with *E. coli* KBN10P07288 induced a robust pro-inflammatory response, as evidenced by significantly elevated plasma concentrations of IL-6 (136.4 ± 11.0 pg/ml), IFN-γ (396.3 ± 42.7 pg/ml), TNF-α (410.3 ± 22.7 pg/ml), and IL-1β (123.4 ± 15.0 pg/ml), compared to uninfected controls (one-way ANOVA, *p* < 0.05) (Fig. 6). Administration of both wild-type and PEGylated phages reduced systemic cytokine levels; however, PEGylated formulations elicited markedly greater anti-inflammatory effects.

Following the first dose, PEG-EC.W2-6 significantly suppressed IL-6, IFN-γ, TNF-α, and IL-1β to 51.2 ± 5.7, 176.2 ± 12.6, 176.2 ± 12.6, and 62.8 ± 4.0 pg/ml, respectively. Comparable reductions were observed for PEG-EC.W15-4 (IL-6: 43.7 ± 12.0; IFN-γ: 173.8 ± 12.0; TNF-α: 173.8 ± 12.0; IL-1β: 57.7 ± 12.0 pg/ml). Notably, cytokine suppression was sustained after the second injection (Day 10), with PEG-EC.W2-6 and PEG-EC.W15-4 further decreasing IFN-γ to 136.8 ± 16.2 and 134.1 ± 11.6 pg/ml, and TNF-α to 146.8 ± 16.2 and 124.1 ± 11.6 pg/ml, respectively. IL-6 levels remained near basal levels, while IL-1β showed a continued downward trend post-treatment.

## Discussion

The therapeutic potential of phages for MDR bacterial infections has long been recognized, yet translation to systemic use has been hindered by rapid immune clearance and poor PK [22, 23]. Most intraperitoneally delivered phages are eliminated within hours due to opsonization, complement activation, and reticuloendothelial uptake [24]. In this study, we demonstrate that surface PEGylation of two structurally distinct phages, EC.W2-6 (*Gordonclarkvirinae*) [3] and EC.W15-4 (*Straboviridae*) [25], substantially improves serum stability, systemic persistence, and therapeutic efficacy in a murine model of MDR *E. coli* ST131 infection. These findings highlight polymer conjugation as a practical strategy to overcome key pharmacological barriers to systemic phage therapy while preserving antibacterial potency.

Although the fluorescamine assay provides only an indirect estimate of amine modification, its reproducibility across independent reactions supports consistent PEG conjugation [17]. The observed reduction in PFU primarily reflects receptor-site shielding rather than particle heterogeneity or structural disruption, as kinetic parameters were unaffected [9]. Future studies employing cryo-TEM, MALDI-TOF, or ^1^H-NMR will be valuable for quantifying PEG grafting density, spatial distribution, and batch-to-batch variability to refine PEGylation control and reproducibility [26].

PEGylation was optimized by titrating PEG-to-amine ratios until saturation of surface-exposed primary amines, avoiding over-conjugation that could impair infectivity by masking RBPs [9]. While PEGylation modestly reduced adsorption kinetics, neither latent period nor burst size was affected, indicating that replication competency was preserved, consistent with earlier reports [17, 27].

In both mouse and human serum, PEGylated phages retained detectable titers beyond 24 h, in contrast to wild-type phages, which rapidly declined. Because heat-inactivated serum was used in these assays, complement activity was absent; therefore, the results primarily reflect intrinsic physicochemical stability rather than immune-mediated clearance. Complement is known to play a central role in rapid *in vivo* phage elimination through opsonization and lysis, and the observed stability likely underestimates the clearance that would occur under complement-active conditions. Nevertheless, the findings support PEG’s protective role against proteolytic and physicochemical degradation, and previous studies implicate complement as a major clearance mechanism [28], suggesting that PEGylation may provide even greater benefit under complement-intact conditions. Future studies incorporating complement-active serum or ex vivo plasma will be important to evaluate the full immunological contribution to phage persistence.

PEGylated phages also exhibited prolonged intracellular stability in RAW264.7 macrophages and HT-29 epithelial cells, maintaining 2–3 log_10_ PFU/ml higher titers than wild-type at 24 h. This suggests enhanced evasion of phagolysosomal degradation, echoing findings for PEGylated nanoparticles that escape lysosomal clearance [29]. These observations confirm that PEGylation prolongs phage half-life in both extracellular and intracellular compartments [9].

PK analyses showed consistent improvements across both phages, including delayed Tmax, increased Cmax, higher AUC, and markedly reduced clearance rates. While wild-type phages typically exhibited rapid elimination and low systemic exposure [30], PEGylated EC.W2-6 and EC.W15-4 demonstrated prolonged circulation with 10–100-fold higher plasma titers. In infected mice, phage amplification occurs selectively in the presence of susceptible *E. coli* hosts, whereas elimination predominates once bacterial loads decline. Because noncompartmental analysis assumes passive elimination, the calculated PK parameters (CL and t_1/2_) should therefore be interpreted as apparent estimates that integrate both replication during active infection and clearance following bacterial eradication. Accordingly, these values represent composite indicators of overall systemic persistence rather than true elimination kinetics.

Even after repeated dosing on day 10, PEGylated phages maintained superior PK profiles despite partial IgG induction. These findings suggest that PEGylation converts short-lived phages into long-acting agents suitable for systemic applications.

In infected mice, PEGylated phages achieved plasma titres several orders of magnitude higher than wild type and maintained them for longer. This translated into faster bacterial clearance, with complete elimination by 72 h compared to 96 h for wild-type phages. PEGylated phages also maintained significantly lower blood CFU counts after both injections, confirming superior therapeutic benefit. Overall, these findings establish that PEGylation enhances both systemic persistence and therapeutic efficacy in the presence of active infection.

Cytokine profiling further revealed that PEGylated phages exert immunomodulatory effects. Systemic *E. coli* infection induced substantial increases in IL-6, IFN-γ, TNF-α, and IL-1β. PEGylated phages significantly reduced these cytokines, surpassing the effects of wild-type phages. After two injections, IFN-γ and TNF-α levels approached baseline, while IL-6 and IL-1β also declined markedly. This suggests that PEGylation enhances not only bacterial clearance but also resolution of infection-associated inflammation [31]. These effects likely stem from both accelerated pathogen elimination and steric shielding by PEG, which limits immune recognition of phage capsids [32]. Together, these mechanisms mitigate the cytokine storm often associated with systemic infection.

A key concern in phage therapy is the generation of neutralizing antibodies [33]. Here, PEGylated phages induced significantly lower anti-phage IgG levels than wild-type phages in both infected and uninfected mice, consistent with PEG’s ability to obscure epitopes from B-cell recognition [34]. Despite some IgG induction, PEGylated phages retained their pharmacological advantage after repeated dosing. However, given reports of anti-PEG antibodies in humans [35], future work should consider alternative stealth strategies such as poly(2-oxazoline) or zwitterionic coatings [36, 37].

This study focused on two previously characterized phages with defined genomic and functional profiles [3, 25]. Wild-type and PEGylated variants were directly compared under identical *in vivo* conditions, allowing for robust assessment of therapeutic performance. Nevertheless, further studies involving complement-active serum and a broader panel of phages and bacterial strains are warranted to validate the generalizability of this approach.

PEGylation is a clinically established methods with multiple FDA-approved applications [38]. Its safety, scalability, and regulatory familiarity position it well for phage therapy. By improving circulation time, reducing immunogenicity, and suppressing pro-inflammatory cytokines, PEGylation addresses fundamental limitations in phage pharmacology, paving the way for clinical translation.

## Conclusion

This study provides compelling evidence that PEGylation is an effective and broadly applicable strategy to overcome the PK and immunological barriers limiting systemic phage therapy. By covalently attaching 5 kDa PEG chains to the capsids of two structurally distinct *E. coli* phages, we achieved >60% surface modification without impairing replication competence. Although adsorption kinetics were modestly slowed, latent period and burst size remained unchanged, confirming that PEGylation preserves lytic potential. Importantly, PEGylated phages displayed markedly enhanced extracellular stability in mouse and human serum, as well as superior intracellular persistence within macrophages and epithelial cells, indicating protection from proteolytic degradation and phagolysosomal clearance. *In vivo*, PEGylation conferred striking PK improvements, including prolonged circulation, reduced clearance, and dramatically increased systemic exposure in both naïve and infected mice. These enhancements translated into accelerated bacterial clearance and robust suppression of inflammatory cytokines, with PEGylated phages inducing weaker IgG responses than wild-type counterparts. Moreover, PEGylated phages effectively suppressed infection-driven cytokines, reducing IL-6, TNF-α, IFN-γ, and IL-1β levels toward baseline while inducing weaker IgG responses. Collectively, these findings establish PEGylation as a practical and scalable approach to enhance systemic phage therapy against MDR pathogens in both naïve and infected mice.

## Supplemental Materials

Supplementary data for this paper are available on-line only at http://jmb.or.kr.



## Figures and Tables

**Table 1:** PK parameters of wild-type and PEGylated bacteriophages in non-infected mice.

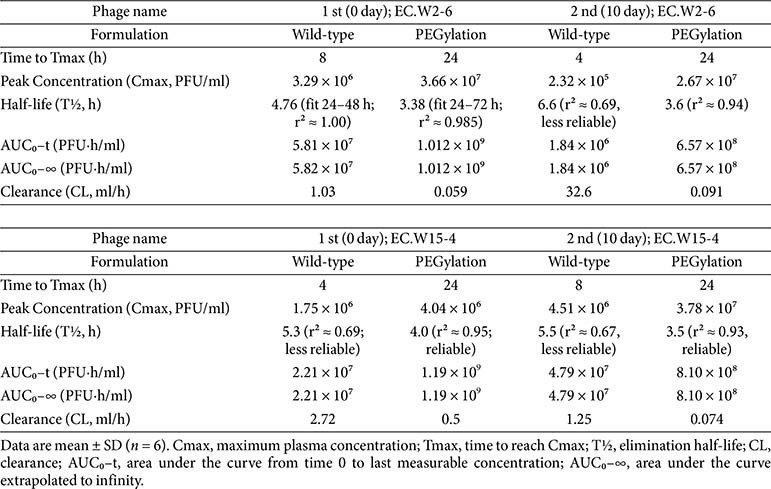

**Table 2:** PK parameters of wild-type and PEGylated phages in infected mice.