# Supplementary material for: PEGylation Overcomes Pharmacological Barriers to Improve Systemic Pharmacokinetics and Therapeutic Efficacy of Phages against MDR Escherichia coli
Source: J Microbiol Biotechnol. 2025 Nov 26;35:e2509050. doi: 10.4014/jmb.2509.09050 (PMC12685592; doi:10.4014/jmb.2509.09050)
Supplement: Supplementary file 1 [file jmb-35-e2509050-supple.pdf]

J. Microbiol. Biotechnol. 2025. 35: e2509050

https://doi.org/10.4014/jmb.2509.09050

Supplementary Figures

PEGylation overcomes pharmacological barriers to improve systemic pharmacokinetics and therapeutic efficacy of phages against MDR *Escherichia coli*

Md Shamsuzzaman[1], Yoon-Jung Choi[1], Shukho Kim[1,2], Ji Yun Jeong[3], Cheol Am Hong[3] and Jungmin Kim[1,2]*

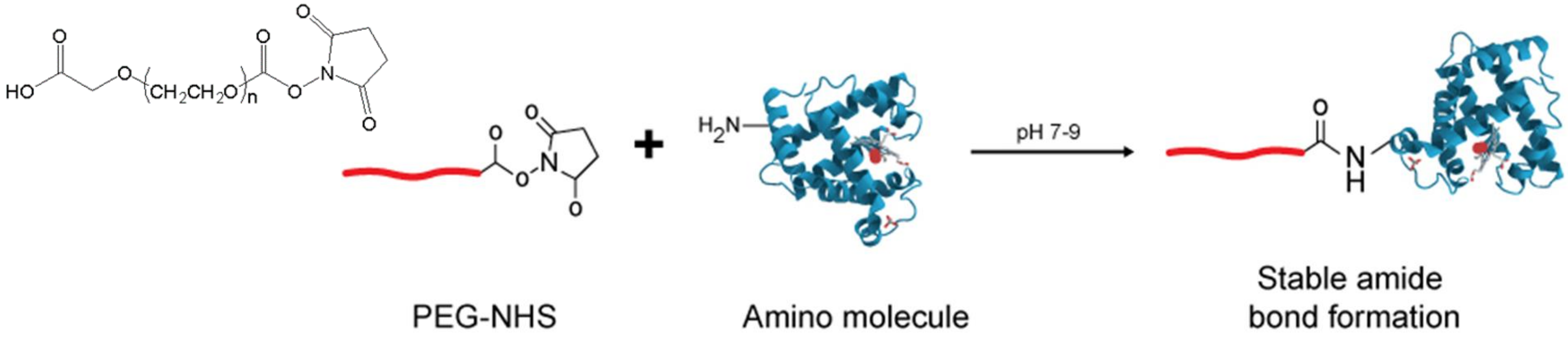

Fig. S1. Schematic representation of amine group conjugation with PEG-NHS. PEG-NHS reacts with primary amines at pH 7–9, forming a stable amide bond commonly used for bioconjugation and protein modification.

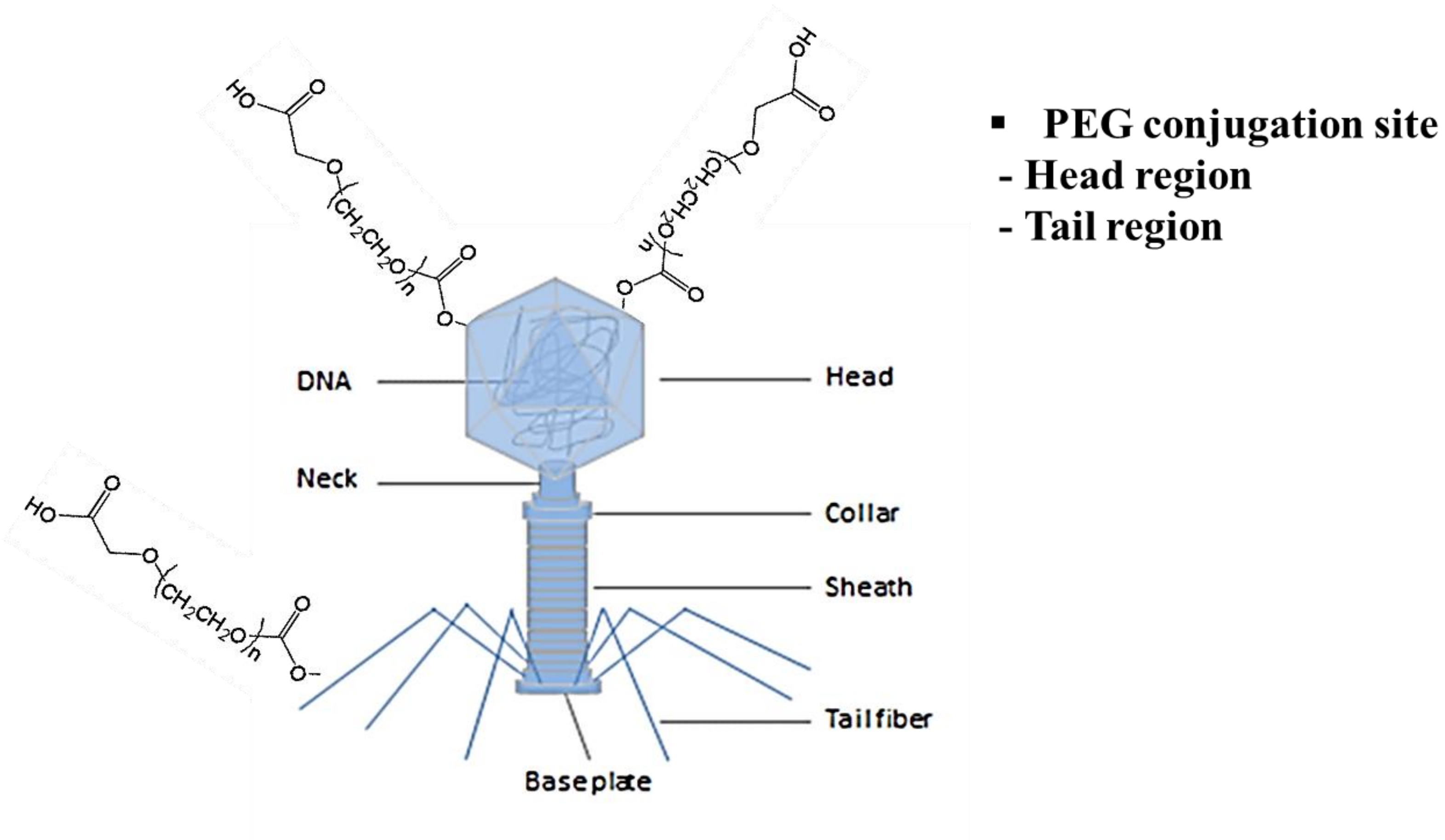

Fig. S2. Schematic representation of PEG conjugation sites on a bacteriophage capsid. PEG chains are covalently attached to amine groups on the phage surface proteins, forming stable PEG–protein linkages.

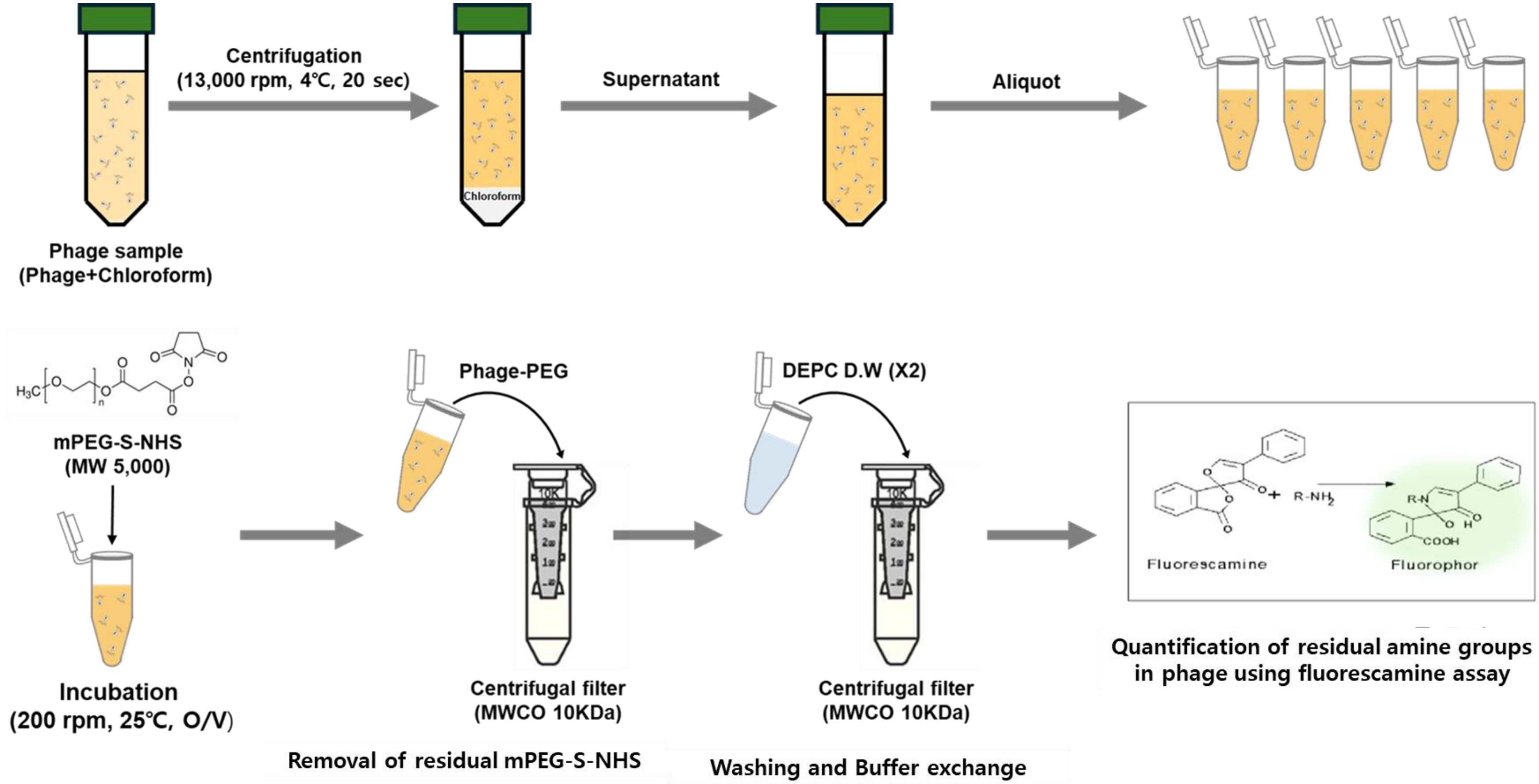

Fig. S3. Schematic workflow for PEGylation of bacteriophages and quantification of residual amine groups. Bacteriophages were conjugated with methoxy polyethylene glycol succinimidyl ester (mPEG-S-NHS), followed by chloroform extraction and multiple washing/buffer exchange steps to remove unreacted PEG. The extent of PEGylation was assessed by quantifying residual free amine groups on phage capsid proteins using a fluorescamine-based fluorescence assay.
